# Digital Therapeutics Care Utilizing Genetic and Gut Microbiome Signals for the Management of Functional Gastrointestinal Disorders: Results From a Preliminary Retrospective Study

**DOI:** 10.3389/fmicb.2022.826916

**Published:** 2022-03-21

**Authors:** Shreyas V. Kumbhare, Patricia A. Francis-Lyon, Dashyanng Kachru, Tejaswini Uday, Carmel Irudayanathan, Karthik M. Muthukumar, Roshni R. Ricchetti, Simitha Singh-Rambiritch, Juan Ugalde, Parambir S. Dulai, Daniel E. Almonacid, Ranjan Sinha

**Affiliations:** ^1^Digbi Health, Mountain View, CA, United States; ^2^Health Informatics, University of San Francisco, San Francisco, CA, United States; ^3^Universidad del Desarrollo, Facultad de Ingeniería, Centro de Investigación en Tecnologías para la Sociedad (C+), Santiago, Chile; ^4^Division of Gastroenterology, University of California, San Diego, San Diego, CA, United States

**Keywords:** multi-omic models, functional gastrointestinal disorders (FGIDs), IBS – irritable bowel syndrome, diarrhea, constipation, digital therapeutics, non-pharmacological treatment

## Abstract

Diet and lifestyle-related illnesses including functional gastrointestinal disorders (FGIDs) and obesity are rapidly emerging health issues worldwide. Research has focused on addressing FGIDs via in-person cognitive-behavioral therapies, diet modulation and pharmaceutical intervention. Yet, there is paucity of research reporting on digital therapeutics care delivering weight loss and reduction of FGID symptom severity, and on modeling FGID status and symptom severity reduction including personalized genomic SNPs and gut microbiome signals. Our aim for this study was to assess how effective a digital therapeutics intervention personalized on genomic SNPs and gut microbiome signals was at reducing symptomatology of FGIDs on individuals that successfully lost body weight. We also aimed at modeling FGID status and FGID symptom severity reduction using demographics, genomic SNPs, and gut microbiome variables. This study sought to train a logistic regression model to differentiate the FGID status of subjects enrolled in a digital therapeutics care program using demographic, genetic, and baseline microbiome data. We also trained linear regression models to ascertain changes in FGID symptom severity of subjects at the time of achieving 5% or more of body weight loss compared to baseline. For this we utilized a cohort of 177 adults who reached 5% or more weight loss on the Digbi Health personalized digital care program, who were retrospectively surveyed about changes in symptom severity of their FGIDs and other comorbidities before and after the program. Gut microbiome taxa and demographics were the strongest predictors of FGID status. The digital therapeutics program implemented, reduced the summative severity of symptoms for 89.42% (93/104) of users who reported FGIDs. Reduction in summative FGID symptom severity and IBS symptom severity were best modeled by a mixture of genomic and microbiome predictors, whereas reduction in diarrhea and constipation symptom severity were best modeled by microbiome predictors only. This preliminary retrospective study generated diagnostic models for FGID status as well as therapeutic models for reduction of FGID symptom severity. Moreover, these therapeutic models generate testable hypotheses for associations of a number of biomarkers in the prognosis of FGIDs symptomatology.

## Introduction

### Background and Rationale

Diseases of the gastrointestinal tract affect 60–70 million people in the United States (Everhart, 2008), and the total expenditure in 2015 for these illnesses was 135.9 billion dollars – greater than for other common diseases and likely to continue increasing (Peery et al., 2019). GI afflictions and altered bowel habits affect the quality of life, social functioning and can result in considerable loss of productivity (Buono et al., 2017; Peery et al., 2019). Thus, early identification of markers to diagnose functional bowel disorders and markers to personalize therapeutic approaches may help reduce healthcare costs and productivity losses and improve quality of life.

According to the National Center for Health Statistics, 73.6% of the US population aged 20 and over was overweight or obese in 2017–2018 (Fryar et al., 2020). Overweight and obesity are known risk factors for the co-occurrence of functional gastrointestinal disorders (FGIDs), and obesity has been shown to negatively impact clinical outcomes of FGID treatment (Emerenziani et al., 2019). Dietary interventions can produce significant weight loss on overweight and obese subjects (Sinha et al., 2021), and at the same time can deliver reduction of FGID symptomatology (Manning and Biesiekierski, 2018).

There is paucity of data looking at demographics, genomic SNPs and microbiome factors that are associated with successfully reducing symptomatology of FGIDs by means of weight loss. Knowledge of these factors could help tailor interventions for these individuals. Thus, our aim for this study was to assess how effective a digital therapeutics intervention personalized on genomic SNPs and gut microbiome signals were at reducing symptomatology of FGIDs on individuals that successfully lost 5% or more body weight. Additionally, we aimed at building statistical diagnostic models to describe the impact of demographics, genomic SNPs, and gut microbiome predictors on the likelihood of a subject presenting or not FGIDs, and then statistical therapeutic models describing reduction of summative symptom severity [irritable bowel syndrome (IBS), diarrhea, constipation, bloating, gas, and abdominal pain] as well as reduction of symptom severity in subjects with IBS, diarrhea, or constipation, based on the same predictors.

### Functional Gastrointestinal Disorders

Functional gastrointestinal disorders are conditions that present as normal upon examination of the GI system but still result in poor GI motility – primarily with symptoms in the middle or lower gastrointestinal tract (American College of Gastroenterology, 2021). These disorders include irritable bowel syndrome (IBS), bloating, constipation, diarrhea, gassiness, and dyspepsia, among others (American College of Gastroenterology, 2021; International Foundation for Gastrointestinal Disorders, 2021). Although the pathophysiology of FGIDs is often complex due to their multifactorial nature, they are frequently encountered in both primary care and gastroenterology settings. FGIDs are thought to encompass combinations of dysregulation of the gut-brain interaction, altered gut microbiota, altered mucosal and immune function, gastrointestinal tract motility disturbance, and visceral hypersensitivity (Black et al., 2020; Sperber et al., 2021), and women appear to be afflicted at least twice as frequently as men (Chang, 2004).

Of the six FGIDs studied here, irritable bowel syndrome (IBS) is the most frequent. It is prevalent in the range of 5–25% of the population and accounts for 36% of visits to a gastroenterologist (Chang, 2004; Buono et al., 2017). It is a gastrointestinal tract disease characterized by abdominal pain, discomfort, altered bowel habits, and affected quality of life, however, the absence of demonstrable organic disease makes IBS to not be considered life-threatening. IBS treatment often requires lifestyle changes and medication. The most common symptoms of IBS are chronic diarrhea – IBS-D (1/3 of IBS patients), chronic constipation – IBS-C, or both - IBS-M. Patients with IBS have a lower reported quality of life than sufferers of gastroesophageal reflux disease (GERD) and asthma (Chang, 2004).

Frequent and recurrent pain in the abdominal region not necessarily attributable to gut function is often referred to as Functional Abdominal Pain (FAP) (Thompson et al., 1999), the second FGID analyzed in this manuscript. Children with FAP were found to be significantly more likely to be obese (Galai et al., 2020). FAP symptoms are not as common as other FGIDs and are not necessarily associated with food intake or passage but rather with psychiatric disorders. Management can involve psychotherapy and pharmaceutical interventions encompassing psychiatric regimens like anti-depressants, anticonvulsants, and treatments for other psychiatric disorders (Clouse et al., 2006).

Functional bloating, the third FGID of focus in this work, on the other hand, is typically unlinked to psychiatric or organic causes (Hasler, 2006). Instead, it is a recurrent sensation of abdominal distention that may or may not be associated with measurable distention. Bloating is 2× more commonly reported in women than men and typically worsens after meals, and up to 96% of IBS patients report bloating (Longstreth et al., 2006; Sullivan, 2012).

Gassiness, the fourth FGID analyzed in this work, is the result of gas produced by bacterial fermentation of carbohydrates and proteins in the large intestine, resulting in changes to the gut microbiome, increased short-chain fatty acids, and increased gas, diarrhea, abdominal pain, and bloating (Hasler, 2006). Although intestinal gas may contribute to bloating, bloating does not necessarily result from more gas. However, malabsorption of simple and complex carbohydrates and dietary fiber are commonly associated with both gas and bloating (Black et al., 2020). Management of gassiness often involves dietary modification, gut microbiome modulation, and lifestyle alteration (Hasler, 2006).

Functional constipation, the fifth FGID studied in our cohort, is a functional bowel disorder that does not meet IBS criteria, and presents as incomplete, infrequent or difficult defecation (Longstreth et al., 2006). Chronic constipation occurs in up to 11% of the population globally and affects all age groups (Sperber et al., 2021). Depending on specific tests, constipation may be defined as (1) straining, hard stools, unproductive movements, infrequent stools, or incomplete evacuation; (2) less than three bowel movements per week OR daily stool weight less than 35 g/day OR straining for more than 25% of the period; (3) lengthy whole-gut or colonic transit. Surprisingly, stool frequency appears to have just a slender relationship with colonic transit and there are usually no demonstrable physiological abnormalities (Longstreth et al., 2006).

Functional diarrhea (FD), the sixth and final FGID analyzed in this work, is a functional gastrointestinal disorder characterized by chronic or recurrent diarrhea not explained by structural or biochemical abnormalities of the gut. Functional diarrhea is characterized as the passage of loose or watery stools without abdominal pain or discomfort (Longstreth et al., 2006). Diarrhea is one of the most commonly reported symptoms for consulting a gastroenterologist, affecting up to 4.7% of the global population (Sperber et al., 2021). It is also a common presenting issue among many patients in general practice (Longstreth et al., 2006). Treatment of FD depends on establishing a correct diagnosis and needs to be distinguished from diarrhea-predominant irritable bowel syndrome (IBS-D) and other organic causes of chronic diarrhea. Once a physician has established the diagnosis, aggravating factors will need to be identified and eliminated, possibly including physiologic factors (e.g., small bowel bacterial overgrowth), psychological factors (e.g., stress and anxiety), and dietary factors (e.g., carbohydrate malabsorption) (Dellon and Ringel, 2006).

Interestingly, it has been hypothesized that FGIDs and obesity may be mechanistically linked (Ho and Spiegel, 2008) and thus that symptom severity for all FGIDs just reviewed may be reduced by means of weight loss through digital therapeutics interventions.

### Association of Diet and Lifestyle With Functional Gastrointestinal Disorders

Diet is considered an important trigger of gut-related symptoms. Poor nutrition, for example, consumption of highly processed and “fast” foods, has been implicated in FGID etiology (Shau et al., 2016; Schnabel et al., 2018). Conversely, a Mediterranean diet has been associated with a lower prevalence of FGIDs (Agakidis et al., 2019). A dietary therapeutics approach, for example a low-FODMAP diet in which rapidly fermentable carbohydrates that are poorly absorbed by the gut are eliminated or avoided as much as possible, is a typical dietary protocol for IBS patients (Henström and D’Amato, 2016; Wilder-Smith et al., 2017). Low-FODMAP diets have been associated with relieving other FGID symptoms, although functional dyspepsia seems least responsive to such a regimen (Marsh et al., 2016; Basnayake et al., 2019).

Dietary fiber, which increases mucosal protein production, is widely used to treat chronic constipation (Derrien et al., 2010). This fiber is then digested in the colon, providing a substrate for microbial fermentation and resulting in byproducts such as Short Chain Fatty Acids (SCFAs), which have pro-motility effects and help with stool bulk and gas transit in the colon (Zhao and Yu, 2016). Moreover, a few reports demonstrate the association of low fiber diet and alterations in gut microbial communities, eventually leading to an increase in FGID symptom severity (Saffouri et al., 2019).

Non-celiac gluten sensitivity also links diet to FGIDs. Despite lacking serological and histological markers for celiac disease, a subset of FGID patients report significant alleviation of symptomatology upon elimination of dietary gluten and re-experience these symptoms upon reintroducing gluten (Elli et al., 2016).

Psychosocial factors have been implicated to varying degrees in FGID etiology, including sleep disturbance, dysfunctional coping, and psychiatric disorders (Longstreth et al., 2006). In particular, sleep disturbance has been demonstrated to be strongly associated with GERD, IBS and functional dyspepsia (Morito et al., 2014). Rotating shift work and poor sleep quality have also been identified as risk factors for IBS (Kim et al., 2013).

A randomized controlled trial by Johannesson et al. (2011) demonstrated that increased physical activity significantly reduced symptom severity in adult IBS patients, leading them to suggest that physical activity should be a primary treatment modality for IBS. Furthermore, a subset of the patients that continued increased physical activity 5 years later, continued to show improvements in IBS symptom severity, as well as in quality of life, fatigue, depression and anxiety in the long term (Johannesson et al., 2015). There is certainly a need for including diet and lifestyle changes in interventions to treat FGIDs.

### Association of Biomarkers With Functional Gastrointestinal Disorders

Both gut microbiome and human genetics are likely contributors to FGID etiology (Henström and D’Amato, 2016; Wei et al., 2021). Evidence indicates a vital genetic component to FGIDs as demonstrated by prevalence within families and more substantial concordance between monozygotic versus dizygotic twins (Lembo et al., 2007; Saito et al., 2010). One evidence of a mutation in the SCN5A gene that regulates the sodium channel, linked to IBS, was replicated across two studies, and although the mutation is found in only approximately 2% of IBS patients, this finding indicates the influence genetics may have on IBS symptoms (Saito et al., 2009; Beyder et al., 2014). Functional constipation has also been associated with specific genes (Locke et al., 2006). However, much of the research around genome-wide association studies and FGIDs is hampered by small sample sizes (Henström and D’Amato, 2016), have not been independently replicated, or are otherwise not robust. Yet, the study of the genetics of FGIDs is rapidly evolving. What seems clear so far is that most IBS sufferers either share several common gene variants that each nominally contribute toward the overall risk of the disease, or, for a subset of sufferers, a few highly penetrant alleles are likely the significant risk factors. Given that IBS spans both complex polygenic conditions and rare single-gene forms, it evidences the need for different strategies to identify these genetic factors.

The gut microbiome is also extensively implicated in FGID and particularly IBS pathogenesis (Agnello et al., 2020; Carco et al., 2020). Both IBS and functional dyspepsia have been shown to arise in susceptible individuals following a course of acute onset gastroenteritis (Simrén et al., 2013). Recent studies have revealed dysbiosis of the gut microbiota in constipated patients compared with healthy controls, associated with suppressed intestinal motility by metabolites produced by intestinal bacteria (Zhao and Yu, 2016; Ohkusa et al., 2019). Other research has elucidated several mechanisms playing important roles in IBS. A dysregulated gut-brain axis has been adopted as a suitable model for IBS, and poor gut microbiome diversity may contribute to the onset and exacerbation of IBS symptoms. Dietary fiber appears to influence the gut microbiota, encouraging the growth of beneficial probiotics while preventing pathogenic and obesogenic bacteria from overgrowing (Chen et al., 2013; Zhao and Yu, 2016). Although clinical trials, which have attempted to characterize the gut microbiota in IBS, do not yet allow for a causal role to be inferred, they do confirm alterations in both community stability and diversity (Kennedy, 2014). This evidence suggests that genomic SNPs and microbiome taxa or functions may help build statistical diagnostic models of the likelihood of a subject presenting or not FGIDs, and therapeutic models describing reduction of FGID symptom severity.

## Materials and Methods

### Subject Enrollment

**FIGURE 1:**
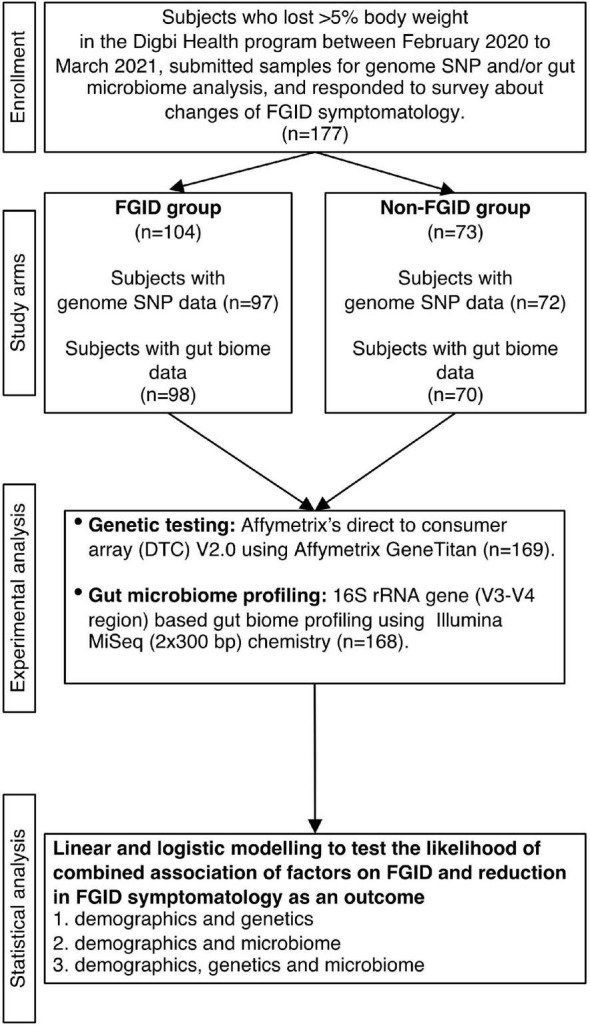
Study design flowchart.

Subjects were recruited from those who achieved 5% or more body weight loss when enrolled in the Digbi Health personalized digital care program (see Intervention below). Only those subjects who had retrospectively responded to questions about symptomatology of their FGIDs and other comorbidities at the start of the program and after successful weight loss were included in the study (Figure 1). The average number of days for participants in the program at the time of the survey was 84.26 days. The final cohort studied in this manuscript included 177 subjects with either genome SNP (*n* = 169) or baseline gut biome (*n* = 168) or both analyses performed. Subjects were divided into two groups for comparisons and models: those who reported any of 6 FGIDs (IBS, diarrhea, constipation, bloating, gassiness, and cramping) at baseline or at the time of survey (*n* = 104), and those who reported no FGIDs (*n* = 73).

### Intervention

Digbi Health is a next-generation, prescription-grade, digital therapeutics platform that analyzes genetics, gut bacteria, lifestyle, and demographics to build evidence-based individualized dietary and lifestyle plans using artificial intelligence (AI). It aims to help subjects reduce at least 5% of their baseline body weight and reduce weight-related inflammatory gut, musculoskeletal, cardiovascular, mental, and insulin-related comorbidities.

Upon enrollment, program participants were provided with online login access to the Digbi Health app and were asked to complete a health questionnaire. A bluetooth compatible digital weighing scale and buccal swab and stool sampling kits were shipped to all participants. The app was used to track subjects’ weight, assess dietary intake (via uploaded photographs of food items consumed), and track wellness and lifestyle associated metrics such as sleep quality and quantity, exercise type and duration, stress and meditation, energy levels, cravings, and recommended foods consumed/avoided. Dietary intake was assessed by coaches who assigned a nutrient density score to meals based on their inflammatory, fiber diversity and expected insulin response.

Based on analysis of genetic and gut microbiome profiles, as well as lifestyle vitals, a Digbi Health Wellness Report was generated for subjects. The results were evaluated with the participants one-on-one by a health coach over the course of 4 months at pre-determined weekly and bi-weekly intervals. To achieve its goal, the program sought to nudge participants toward making incremental lifestyle changes focused on reducing sugar consumption and timing meals to optimize insulin sensitivity, reducing systemic inflammation by identifying possibly inflammatory and anti-inflammatory nutrients, and increasing fiber diversity to improve gut health. Most importantly, these behavioral changes were implemented with the help of virtual health coaching and the app to ensure that these changes are habit forming, i.e., long-term sustainable.

### Sample Collection and Processing: Genome SNP Array and Gut Microbiome Profiling

Subjects self-collected buccal swab samples (Mawi Technologies iSwab DNA collection kit, Model no. iSWAB-DNA-1200) and fecal swab samples (Mawi Technologies iSWAB Microbiome collection kit, Model no. ISWAB-MBF-1200). Sample collection was completed by following standardized directions provided to all subjects in an instruction manual. DNA extraction, purification, and genotyping from buccal swab samples was performed using Affymetrix’s Direct to Consumer Array version 2.0 (“DTC”) on the Affymetrix GeneTitan platform at Akesogen Laboratories in Atlanta, GA, United States. Sample processing of baseline (pre-intervention) fecal samples was followed by 16S rRNA gene amplicon sequencing also performed at Akesogen Laboratories in Atlanta, GA, United States. DNA extraction was performed using Qiagen MagAttract Power Microbiome DNA Kit on an automated liquid handling DNA extraction instrument. The V3–V4 region of the 16S rRNA gene was amplified and sequenced on the Illumina MiSeq platform using 2 × 300 bp paired-end sequencing (Illumina, 2013). Sequence reads were demultiplexed, and Amplicon Sequence Variants (ASVs) generated using DADA2 in QIIME2 (version 2020.8) (Bolyen et al., 2019). We trimmed primers off the reads and low-quality bases (*Q* < 30). Taxonomic annotation was performed using the Naive Bayes classifier against the 99% non-redundant Silva database (Silva 138 Reference database, 2021). We excluded hits to Mitochondria, Chloroplast, Eukaryota, and unassigned taxa at the phylum level.

### Statistical Analysis

Survey data from 177 respondents (with either genome SNP or gut biome data) over the course of their successful weight loss journey in the Digbi Health personalized digital care program were analyzed retrospectively. Variables for models included demographic (gender, age, weight, and BMI during gut microbiome sample collection and weight loss achieved during the program), genomic SNPs, and baseline gut microbiome data. We used the Wilcoxon sum rank/signed-rank test or Kendall correlation test, as appropriate, to assess: (a) the change in the severity of either of the FGID symptoms and a summative severity change and (b) the effect of variables on the change in severity. Significance results were adjusted for multiple comparisons using the false discovery rate (FDR) correction method for the microbiome data.

### Genome SNP Related Statistics

The 16 SNP predictors, associated with lactose intolerance, gluten sensitivity, milk and peanut allergies, caffeine metabolism, and inflammatory markers (TNF and IL10), were from Digbi Health curated panels used for personalized interventions for subjects (Supplementary Table 1). Each SNP value was encoded as the number of risk alleles (0, 1, or 2) for each subject.

### Microbiome Related Statistics

Bacterial genera abundances were analyzed for 168 survey respondents with baseline gut microbiome data available, using Qiime2 (Bolyen et al., 2019), qiime2R (Jordan E Bisanz, 2018), and phyloseq (McMurdie and Holmes, 2013). The following microbial features were filtered out from downstream analysis: (a) ASVs not classified at the phylum level, (b) phyla that had <25 ASVs (*Elusimicrobiota*, *Nanoarchaeota*, *Bdellovibrionota*, WPS-2), (c) uncultured and Incertae Sedis taxa, (d) genera that had <30 reads in at least 15% of samples, and (e) genera for which >25% of samples had zero read count. In total, 105 genera were kept in downstream analysis. The abundance of these bacterial genera was transformed to centered log-ratio (CLR) using the zCompositions package (Palarea-Albaladejo and Martín-Fernández, 2015) after first replacing zeros with pseudo counts based on a Bayesian-multiplicative replacement from the zCompositions package (Fernandes et al., 2014). Permutational multivariate analysis of variance (PERMANOVA) was performed on the gut microbiome Aitchison distance matrix, using gender, BMI (closest to date of gut biome sampling), and FGID status as variables using the CLR transformed abundances. Additionally, the read counts after zero replacement were transformed using additive log-ratio (ALR) to be utilized in downstream model statistics (see below) (Friedman et al., 2010; Gloor et al., 2017; Quinn et al., 2019).

### Model Statistics

Linear and logistic regression models were built, and visualizations generated using the R stats, ggplot2, pscl, car, pROC, Metrics, caret, glmnet, tidyverse, lubridate, imputeTS, and ggpubr packages. In order to utilize lasso regression for variable selection before fitting linear and logistic models, SNPs with >10% missing values were removed, then remaining missing SNPs were imputed to their most frequent value (mode). This resulted in the removal of rs4713586 gluten sensitivity SNP from both reduction in summative symptom severity and reduction in constipation symptom severity models. In order to avoid poor performance in regression models, variables with Pearson correlation to another variable of ≥80% were removed. This excluded two SNPs from all regression models that incorporated DNA: rs182549 of the lactose persistence haplotype was removed while the more highly cited correlated (Pearson correlation, *r* = 0.99) haplotype SNP, rs4988235, was retained. Additionally, IL10 SNP rs3024496 was removed as being highly correlated (Pearson correlation, *r* = −0.95) with rs1800896, another IL10 SNP, which was preferentially retained as having a higher risk in the population (SNPedia database, 2021).

For regression models, we transformed bacterial abundance data using the additive log-ratio (ALR) (Friedman et al., 2010; Gloor et al., 2017), which maintains sub-compositional coherence, permitting genera to be removed in downstream analysis (for example, removing insignificant predictors from a regression model). For this, the easy Coda package (Greenacre, 2018) was employed to analyze all 105 microbial genera utilizing variances, variances explained, and Procrustes correlations to select candidate references for ALR according to methodology of Greenacre et al. (2021). An additional criterion we employed was prevalence, as zero values are problematic with log-ratios. Based on these criteria, *Blautia* was the selected reference. In all cases of highly correlated microbe pairs, the unclassified microbe was removed, or if both were unclassified, then the microbe with the larger mean absolute correlation in that dataset was removed. This resulted in removing up to 5 microbes (of 105) from regression models incorporating microbial predictors. Gender and SNPs were scaled to a range between −1 and 1. Age and gender were the two demographic variables used in all models; age was used in models with no transformation. Our hypothesis was that age and gender would be associated with bacterial taxa as evident from literature and are important factors considered in our personalization and care interventions. Hence, we decided to include gender and age as well in all the models and test their association in the context of the moderation effect of genetic and microbiome variables.

After the above data preparation, lasso was employed (glmnet package), for variable selection in the modeling of datasets that included the 105 microbial predictors. Optimal lambda was chosen by a fivefold cross-validation grid search, setting standardize = TRUE so that variables would compete fairly in regularization. The lambda resulting in the minimum mean cross-validated error was selected, or if this resulted in a paucity of predictors, then a plateau lambda in error vs. lambda plot having a sufficient number of predictors was selected. Mean squared error was employed in linear lasso regression, while the mean absolute error was used in logistic regression models. Predictors with non-zero coefficients were retained for subsequent best-fit regression to describe the FGID differences of the subjects over the course of treatment.

To arrive at these descriptive models the step function (stats package) was employed, using the Akaike information criterion (AIC) to obtain a high-quality fit. If any insignificant variables remained, these were removed one-by-one beginning with the least significant, until only significant variables remained, resulting in a final interpretable model for each investigation and set of predictors.

The descriptive modeling of FGID vs. non-FGID was conducted by fitting a logistic regression model, as described above, to demographic and 14 SNPs genomic data (D + G model), producing coefficients to describe the impact of each predictor on FGID in this cohort. A second logistic model was fit with demographic plus 105 baseline gut microbiome genera remaining after pre-processing (D + M model). A third logistic model similarly employed lasso for variable selection from demographic variables, 14 genomic SNPs, and 104 baseline genera (D + G + M model).

For those respondents who self-reported any of the 6 FGIDs, change in symptom severity was analyzed with respect to their demographic, genome SNPs, and baseline gut microbiome data. 104 survey respondents rated the severity of their FGID symptoms on a scale of 1–5. A linear regression model was fit to describe the change in summative symptom severity as a function of demographic and genomic variables (D + G model). As above, this model was compared with two additional models: demographic plus microbial predictors (D + M model) and demographic, genomic, plus microbial predictors (D + G + M model). Lasso regression was employed as above for variable selection, followed by the use of the step function. Additionally, changes in IBS, diarrhea and constipation symptom severity were modeled for those subsets of participants who reported them, using lasso with threefold cross-validation and linear regression as above with D + G, D + M, and D + G + M predictors.

### Ethics Statement

E&I Review Services, an independent institutional review board, reviewed and approved IRB Study #18053 on 05/22/2018. Additionally, IRB Study #21141 was determined to be exempt from E&I Review Services on 08/06/2021. Research material derived from human participants included self-collected buccal and fecal swabs. Informed consent was obtained electronically from study participants.

## Results

### Subject Characteristics

In total, 177 subjects who were successful at losing 5% or more body weight and had genetic and/or gut biome data while enrolled in the Digbi Health program were surveyed to assess any changes in the symptomatology of their FGIDs (Figure 1). We compared baseline characteristics of those who reported FGIDs vs. those who did not. The distributions of gender, age, and BMI are seen in Table 1. In this dataset, a significant difference was found in gender (*X*^2^_1_ = 8.39, *P* = 0.004) and initial BMI (Wilcoxon sum rank test, *P* = 0.009), between those who reported FGID and those who did not, but no significant difference was found in age (Wilcoxon sum rank test, *P* = 0.60), number of individuals consuming alcohol or using recreational drugs such as cannabinoids and nicotinoids (including tobacco smoking) (*X*^2^_1_ = 2.52, *P* = 0.112 and *X*^2^_1_ = 0.28, *P* = 0.595, respectively). Subsequently, we investigated the effect of gender, BMI, and FGID status on the beta diversity of the baseline gut microbiome of subjects. The PERMANOVA analysis (Supplementary Table 2) shows that gender had a significant effect on the beta diversity (*R*^2^ = 0.012, *P* = 0.030), whereas BMI (*R*^2^ = 0.007, *P* = 0.279) and FGID status (*R*^2^ = 0.005, *P* = 0.532) did not.

**TABLE 1:** Distribution and demographics of FGID and non-FGID groups in the study.

Descriptor variable	FGID group (*n* = 104)	Non-FGID group (*n* = 73)	*P*-value
Gender, *n* (%)			
Male	12 (11.54%)	22 (30.14%)	*0.004*^a^**
Female	92 (88.46%)	51 (69.86%)	
Age, in years [median (*SD*)]	49 (11.92)	51 (12.25)	*0.*60
BMI closest to gut biome sampling [median (*SD*)]	31.17 (7.37)	33.51 (7.50)	*0.009*
Alcohol consumption, *n* (%)	62 (59.61%)	52 (71.23%)	0.112
Recreational drugs (including tobacco smoking), *n* (%)	19 (19.2%)	16 (22.5%)	0.595

*^a^Values in italics are significant (P = < 0.05).*

### Gut Microbiome Taxa Are a Stronger Predictor of Functional Gastrointestinal Disorders Status Than the Genomic SNPs Analyzed in This Study

The SNP values were not significantly different (Welch’s two-sample *t*-tests, results not shown) between respondents with FGID and those without. Logistic regression modeled the associations of demographic, genome SNP, and baseline gut microbiome variables with FGID status in this cohort (Tables 2–4), fitting separate average effect size for each predictor while controlling for all other model variables. The D + G model described females in this cohort as 3.26 times more likely than males to suffer FGID while controlling for the genomic predictors in the model (Table 2), and each risk allele of the rs2187668 gene was associated with a 2.93 times greater likelihood of being an FGID sufferer. Similarly, risk alleles for rs2472297 and rs9275596 were associated with a lowered likelihood of FGID – conferring 0.45 and 0.56 times likelihood of being an FGID sufferer.

**TABLE 2:** Functional gastrointestinal disorders (FGID) vs. non-FGID logistic model: demographics + genomics (D + G).

Variable	OR	2.5% CI	97.5% CI
Gender	3.255	1.421	7.856
Caffeine metabolism (rs2472297), Risk Allele C	0.447	0.211	0.888
Gluten sensitivity (rs2187668), Risk Allele T	2.926	1.140	8.269
Peanut allergy (rs9275596), Risk Allele C	0.556	0.323	0.935

*McFadden pseudo R^2^: 0.089, OR, odds ratio.*

In a second logistic model, we studied the associations of D + M together on the likelihood of a subject having an FGID (Table 3). The nine taxa identified by lasso regression were used as predictors for a logistic regression model to describe the classification of 168 subjects into their corresponding FGID status, using the genus *Blautia* as the reference denominator for ALR. In this model, the effect of the female gender while controlling for microbial predictors was an average 2.33 times likelihood of FGID compared with the male gender. Genera *Ruminococcus torques* group, *Akkermansia*, unclassified genus CAG-56 of Lachnospiraceae family, *Haemophilus*, and *Terrisporobacter* were all associated with increased FGID. Whereas *Holdemanella*, unclassified genus UCG-010 of *Oscillospirales* order, *Anaerostipes*, and *Fusicatenibacter* were all associated with decreased (protective of) FGID status.

**TABLE 3:** Functional gastrointestinal disorders vs. non-FGID logistic model: demographics + microbiome (D + M).

Variable	OR	2.5% CI	97.5% CI
Gender	2.334	1.213	4.686
*Ruminococcus torques* group	1.193	1.039	1.391
*Akkermansia*	1.076	1.011	1.151
*Holdemanella*	0.932	0.878	0.986
Unclassified genus CAG-56 of *Lachnospiraceae* family	1.095	1.024	1.175
Unclassified genus UCG-010 of *Oscillospirales* order	0.904	0.839	0.970
*Anaerostipes*	0.806	0.680	0.938
*Haemophilus*	1.115	1.034	1.210
*Fusicatenibacter*	0.894	0.803	0.985
*Terrisporobacter*	1.076	1.008	1.153

*McFadden pseudo R^2^: 0.220, OR, odds ratio.*

In a third logistic model studying the associations of D + G + M variables together on the likelihood of a subject having FGID status (Table 4), no SNPs had a significant association with FGID risk. Variables in Tables 3, 4 are identical, with just slight variations in odd ratios. Not surprisingly, pseudo *R*^2^ values from the D + M model (0.220) and the D + G + M model (0.227) were very similar, but most importantly, improved from that of the D + G model (0.089).

**TABLE 4:** Functional gastrointestinal disorders vs. non-FGID logistic model: demographics + genomics + microbiome (D + G + M).

Variable	OR	2.5% CI	97.5% CI
Gender	2.256	1.151	4.638
*Ruminococcus torques* group	1.186	1.030	1.388
*Akkermansia*	1.080	1.013	1.156
*Holdemanella*	0.928	0.871	0.985
Unclassified genus CAG-56 of *Lachnospiraceae* family	1.100	1.028	1.184
Unclassified genus UCG-010 of *Oscillospirales* order	0.912	0.845	0.978
*Anaerostipes*	0.797	0.671	0.929
*Haemophilus*	1.098	1.017	1.193
*Fusicatenibacter*	0.892	0.801	0.983
*Terrisporobacter*	1.074	1.003	1.153

*McFadden pseudo R^2^: 0.227, OR, odds ratio.*

### Subjects Reported a Reduction in Severity of Functional Gastrointestinal Disorders Related Symptoms Over the Course of Treatment

**FIGURE 2:**
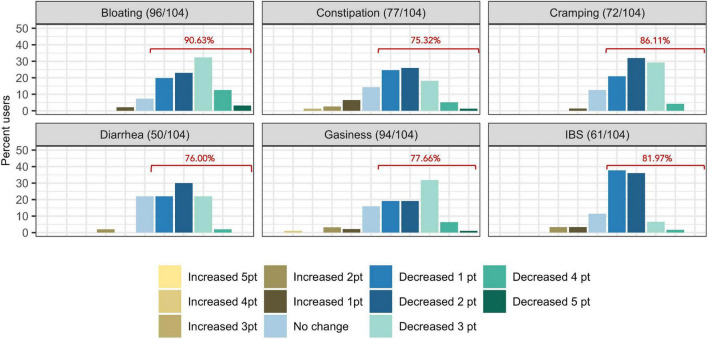
Self-reported symptom severity change (number of levels increased/decreased severity) in functional gastrointestinal disorders from baseline to time of survey. Percentages displayed in red indicate the proportion of users who reported at least one point reduction is symptom severity.

The proportion of subjects who experienced at least one point improvement in symptom severity ranged from 75.32% (77/104) for constipation to 90.63% (96/104) for bloating (Figure 2). Improvement in summative severity across the 6 FGIDs was seen by 89.42% of respondents (93/104), with an average summative reduction of 51.17% (Wilcoxon signed-rank test, *P* = <0.001). The improvement in FGID symptomatology over the course of the digital therapeutics intervention (percent summative reduction) was not correlated with percent weight loss (Kendall = 0.12, *P* = 0.91), age (Kendall = −0.66, *P* = 0.51), or gender (Wilcoxon sum rank, *P* = 0.809). Individually, we noted an average 45.93% reduction in the severity of IBS (Wilcoxon signed-rank, *P* = <0.001), 61.01% average reduction in the severity of bloating (Wilcoxon signed-rank test, *P* = <0.001), 38.55% average reduction in the severity of gassiness (Wilcoxon signed-rank, *P* =< 0.001), 61.69% average reduction in the severity of cramping/belly pain (Wilcoxon signed-rank, *P* = <0.001), 37.54% average reduction in the severity of constipation (Wilcoxon signed-rank, *P* = <0.001) and a 48.97% average reduction in the severity of diarrhea (Wilcoxon signed-rank, *P* = <0.001).

### Genomic and Microbiome Predictors Can Model Reduction in Summative Functional Gastrointestinal Disorders Symptom Severity as a Result of the Digital Therapeutics Intervention

Supplementary Tables 3, 4 and Table 5 present linear D + G, D + M, and D + G + M models of reduction in summative FGID symptom severity, respectively. In the D + G model (Supplementary Table 3), each additional risk allele for SNPs rs4639334 and rs7775228 was associated with an increase in self-reported summative FGID symptom severity. In the linear D + M model (Supplementary Table 4), gut microbial genera Candidatus *Soleaferrea*, *Eubacterium hallii* group, *Alistipes*, and *Desulfovibrio* were all associated with an increase in self-reported summative FGID symptom severity. Microbial genera *Ruminococcus torques* group, *Intestinimonas*, unclassified genus GCA-900066575 of Lachnospiraceae family, and *Megasphaera* were all associated with a self-reported reduction in summative FBD symptom severity. In the linear D + G + M model (Table 5), risk alleles of the same two SNPs identified (Supplementary Table 3) were again associated with an increase in self-reported summative FGID symptom severity. Similarly, gut microbial genera *Desulfovibrio*, Candidatus *Soleaferrea*, and *Eubacterium ventriosum* group were associated with self-reported increase in summative FGID symptom severity. Microbial genera *Megasphaera*, unclassified genus CAG-352 of Ruminococcaceae family, *Ruminococcus torques* group, *Streptococcus*, and *Intestinimonas* were all associated with self-reported reduction in summative FGID symptom severity. Adjusted *R*^2^ values were 0.124 for the D + G model, 0.318 for the D + M model, and 0.442 for the D + G + M model. This indicates that the fit of the models improved when adding microbiome predictors, with the best fit model for a reduction in summative FGID symptom severity containing a mixture of genomic SNP and microbiome variables.

**TABLE 5:** Reduction in summative FGID symptom severity linear model: demographics + genomics + microbiome (D + G + M).

Variable	Estimate	Std. error	*t*-value	*P*-value
*Megasphaera*	0.495	0.136	3.631	<*0.001*^a^
*Desulfovibrio*	−0.517	0.105	−4.910	<*0.001*
Unclassified genus *CAG-352* of *Ruminococcaceae* family	0.257	0.104	2.477	*0.015*
Gluten Sensitivity (rs4639334), Risk Allele A	−2.013	0.792	−2.541	*0.013*
*Ruminococcus torques* group	0.646	0.246	2.626	*0.010*
Gluten Sensitivity (rs7775228), Risk Allele C	−1.936	0.828	−2.338	*0.022*
*Streptococcus*	0.505	0.218	2.322	*0.023*
*Intestinimonas*	0.319	0.116	2.756	*0.007*
Candidatus *Soleaferrea*	−0.461	0.150	−3.076	*0.003*
*Eubacterium ventriosum* group	−0.290	0.134	−2.157	*0.034*

*Adjusted R^2^: 0.442.*

*^a^Values in italics are significant (P = <0.05).*

### In the Descriptive Models, Reduction in Irritable Bowel Syndrome Symptom Severity Was Better Explained by a Mixture of Genomic and Microbiome Predictors, Whereas Reduction in Diarrhea and Constipation Symptom Severity Was Better Explained by Microbiome Predictors Only

Tables 6–8 present linear D + G + M models for reduction of symptom severity for IBS, constipation, and diarrhea, respectively. Supplementary Tables 5, 7, and 9, present linear D + G models, and Supplementary Tables 6, 8, and 10, present linear D + M models for reduction of symptom severity for IBS, constipation, and diarrhea, respectively. Adjusted *R*^2^ values for the reduction in IBS symptom severity models were 0.130 for the D + G model, 0.432 for the D + M model, and 0.487 for the D + G + M model. Adjusted *R*^2^ values for the reduction in constipation symptom severity models were 0.038 for the D + G model, 0.413 for the D + M model, and 0.389 for the D + G + M model. Adjusted *R*^2^ values for the reduction in diarrhea symptom severity models were 0.090 for the D + G model, 0.610 for the D + M model and 0.528 for the D + G + M model. This shows that genomic SNP models based on the variables selected for this study performed relatively poorly and that the inclusion of microbiome variables constantly improved the fit of the models. Interestingly, the D + G + M was the best fit model for IBS symptom reduction, whereas, for constipation and diarrhea symptom reduction, the D + M models were the best fit.

**TABLE 6:** Reduction in IBS symptom severity linear model: demographics + genomics + microbiome (D + G + M).

Variable	Estimate	Std. error	*t*-value	*P*-value
Gluten sensitivity (rs7775228), Risk Allele C	−0.566	0.225	−2.515	*0.016*^a^**
Unclassified genus *Clostridia UCG-014*	0.092	0.024	3.844	<*0.001*
*Escherichia-Shigella*	0.087	0.028	3.122	*0.003*
*Fusicatenibacter*	−0.096	0.038	−2.501	*0.016*
*Megasphaera*	0.101	0.034	3.007	*0.004*
*Moryella*	−0.079	0.028	−2.775	*0.008*

*Adjusted R^2^: 0.487.*

*^a^Values in italics are significant (P = <0.05).*

Looking at the best fit models, Unclassified genus *Clostridia UCG-014*, *Escherichia-Shigella*, and *Megasphaera* were associated with reduction of IBS symptom severity, whereas risk alleles of rs7775228 (gluten sensitivity) and genera *Fusicantenibacter* and *Moryella* were associated with an increase. For constipation, there were only microbial taxa in the D + M model: *Parabacteroides*, Unclassified genus of *Anaerovoracaceae* Family XIII AD3011 group, *Lachnospira* and *Terrisporobacter* were associated with a reduction in symptom severity, whereas *Eubacterium coprostanoligenes* group was associated with an increase. For diarrhea change in symptom severity, like for constipation, only microbial taxa were found significant in the D + M model: genera *Intestinimonas*, *Prevotella*, *Lactobacillus*, and *Phascolarctobacterium* were associated with a reduction in symptom severity, while Unclassified genus UCG-009 of the *Butyricicoccaceae* family was associated with an increase.

## Discussion

Out of 177 subjects enrolled in this study who successfully lost 5% or more body weight through a digital therapeutics program, 104 presented one or more FGIDs. These FGID sufferers were significantly different from the non-FGID group in terms of gender and BMI at the time of sampling (Table 1). Additionally, gender was significantly associated with the composition of baseline gut microbiome samples in these subjects (Supplementary Table 2). Logistic regression models trained to differentiate FGID status confirmed that the female gender was associated with a higher prevalence of FGID as seen in the logistic regression D + G model, where females were 3.26 times more likely to be FGID sufferers than males while holding genomic predictors constant (Table 2). This is in accordance with what has been reported elsewhere (Narayanan et al., 2021). Additionally, when baseline microbiome was added to the model (D + G + M model), females were on average 2.26 times more likely to suffer from FGID than males while holding constant both genomic and microbial predictors (Table 4). Thus, some of the gender association in the D + G model is explained by microbiome variables in the D + G + M model, reinforcing the role of gender in shaping the baseline gut microbiome of subjects (Kim et al., 2020).

Of note, the SNP: gluten sensitivity (rs2187668, risk allele T) was seen to be strongly associated with FGID status in our cohort. This SNP variant is identified as HLA-DQ2.5 and has been reported as one of the most common HLA-DQ2 haplotypes associated with celiac disease (Van Heel et al., 2007). Interestingly, the D + G + M model did not select any genomic variables and was identical to the D + M model (same variables but slightly different odd ratios), and not surprisingly, the pseudo *R*^2^ scores for these models are similar (0.227 for D + G + M vs. 0.220 for D + M). These models have pseudo *R*^2^ scores higher than for the D + G model (0.089), indicating that baseline microbiome better classified participants having FGID than models based on genomic predictors and that the addition of SNPs did not improve classification of FGID by gender plus baseline microbiome. Many of the microbiome taxa variables identified in the models have already been reported in the literature associated with FGIDs. For instance, previous studies show a strong association of the *Ruminococcus torques* group with FGIDs (Lyra et al., 2009).

We then investigated the change in reported symptom severity over the course of the digital therapeutics program. In total, 89.42% (93/104) of subjects experienced improvement in summative symptom severity of their reported FGIDs. This improvement in symptom severity was not correlated with percent weight loss, gender, or age. Reduction in symptom severity was significant for all six FGIDs investigated individually and for summative symptom severity (all of them together). When we modeled the reduction in summative FGID symptom severity over the course of digital therapeutics intervention, we identified the D + G + M linear model as the best fitting, with an adjusted *R*^2^ of 0.442, compared with 0.124 for the D + G model and 0.318 for the D + M model. Two genomic SNPs and eight microbial taxa were the significant predictors in the best model for these participants. Thus, in our cohort, gender, and baseline microbiome best classified subjects into their FGID status, whereas a combination of genomic SNPs and microbiome variables (but not gender) best-modeled reduction in summative FGIDs symptom severity.

We then looked at the reduction of symptom severity for three functional bowel disorders of our interest: IBS, constipation, and diarrhea. Interestingly, for IBS, the best fit model was the one containing D + G + M variables, whereas, for constipation and diarrhea, the best fit models were those only containing D + M variables.

When analyzing the variables found significant to the best fit models for this cohort, we identified several genomic SNPs and microbial taxa that are shared across two or more models. SNP rs7775228 (gluten sensitivity; risk allele C) was associated in the linear D + G + M model with an increase in summative symptom severity (Table 5), as well as in the linear D + G + M model with an increase in IBS symptom severity (Table 6). Interestingly, despite not being the best fit model, it also was associated in the linear D + G model with an increase in diarrhea symptom severity (Supplementary Table 9) and associated in the linear D + G + M model with reduction of constipation symptom severity (Table 7). In addition to its association with gluten sensitivity (Monsuur et al., 2008), which is why we included this SNP as part of our care protocols, rs7775228 is involved in seasonal allergic rhinitis and as a protein biomarker for inflammation (Monsuur et al., 2008; Enroth et al., 2014).

**TABLE 7:** Reduction in constipation symptom severity linear model: demographics + genomics + microbiome (D + G + M).

Variable	Estimate	Std. error	*t*-value	*P*-value
*Parabacteroides*	0.231	0.073	3.174	*0.003*^a^**
*Eubacterium coprostanoligenes* group	−0.161	0.051	−3.125	*0.003*
*Lachnospira*	0.123	0.047	2.606	*0.013*
Gluten sensitivity (rs7775228), Risk Allele C	0.757	0.327	2.311	*0.026*

*Adjusted R^2^: 0.389.*

*^a^Values in italics are significant (P = <0.05).*

In terms of the microbial taxa shared across two or more models, genus *Fusicatenibacter* was associated with decreased likelihood of having FGID status in the D + G + M logistic model of this cohort (Table 4) and associated with an increase in IBS symptom severity in the D + G + M linear model (Table 6). Genus *Intestinimonas* was associated with a reduction in summative FGID symptom severity in the D + G + M model (Table 5) and a reduction in diarrhea symptom severity in the D + M model (Supplementary Table 10). Genus *Megasphaera* was noted to be strongly associated with a reduction in summative FGID symptom severity in the D + G + M linear model of Table 5 and a reduction in IBS symptom severity in the D + G + M linear model of Table 6. Interestingly, genus *Lactobacillus* appears associated with a reduction in diarrhea symptom severity in the linear D + M model (Supplementary Table 10), an effect that has been amply demonstrated in the literature (McFarland and Goh, 2019), supporting the validity of our methods. Moreover, these bacteria, specifically, *Fusicatenibacter*, *Intestinimonas*, and *Megasphaera* have been previously reported to be Short Chain Fatty Acids (SCFAs) producers (Takeshita et al., 2016; Jin et al., 2019; Bui et al., 2020; Luu et al., 2021). There is ample evidence of the role of SCFAs in improving gut integrity, which plays an essential role in maintaining mucosal homeostasis (Tan et al., 2014), and may explain why these taxa were either negatively associated with FGID status or were associated with a reduction in the severity of FGID symptoms in our cohort.

Our analysis also revealed some bacterial taxa that were associated with FGID status. *Desulfovibrio* was observed to possess a significant association with an increase in FGID summative symptom severity (Table 5). Previous reports suggest that bacteria belonging to the genus *Desulfovibrio* generate H2S gas via a dissimilatory sulfate reduction pathway, leading to inflammatory gut disorders (Singh and Lin, 2015; Kushkevych et al., 2019). We also noted the association of *Akkermansia* with increased FGID likelihood (Table 4). Although this bacterium is suggested to have beneficial associations with gut health, a few studies have reported its inverse correlation with reduction of abdominal pain (Cruz-Aguliar et al., 2019).

*Ruminococcus torques* group appears associated with FGID status in the D + G + M logistic model of Table 4 and associated with a reduction in summative FGID symptom severity in the D + G + M model of Table 5. Despite not being the best fit model, this taxon also was associated with a reduction of diarrhea symptom severity in the linear D + G + M model of Table 8. Different *Ruminococcus torques* subgroups have been associated in the literature with IBS-D, IBS-M, and Crohn’s disease subjects (Lyra et al., 2009). Additionally, genus *Terrisporobacter*, associated with inflammation and gut dysbiosis (Lee et al., 2020), was associated with FGID status in the D + G + M logistic model (Table 4). Collectively, these findings demonstrate the potential of gut microbial profiling not only for predicting current gastrointestinal health but also prognosis in FGID related symptoms as a response to personalized dietary intervention.

**TABLE 8:** Reduction in diarrhea symptom severity linear model: demographics + genomics + microbiome (D + G + M).

Variable	Estimate	Std. error	*z*-value	*P*-value
*Ruminococcus torques* group	0.203	0.076	2.669	*0.013*^a^**
Lactobacillus	0.114	0.042	2.706	*0.012*
Unclassified genus UCG-009 of *Butyricicoccaceae* family	−0.107	0.035	−3.045	*0.005*
Prevotella	0.069	0.031	2.249	*0.034*

*Adjusted R^2^: 0.528.*

*^a^Values in italics are significant (P = <0.05).*

### Limitations

This study has some limitations that are important to note. First, the descriptive modeling exercise performed in this work is the best fit for the cohort analyzed here and is not intended to infer for the larger population. In particular, we aimed to investigate which demographic, genomic, and baseline microbiome predictors improved the fit of the models, along with the magnitude and direction of their association with FGID status or symptom prognosis. Second, whereas we used all microbiome taxa present in the baseline gut microbiome samples (*n* = 105 after the filters imposed), for genomic SNPs, we selected only markers that are used to inform diet and lifestyle interventions of subjects under the Digbi Health program, specifically those associated with intolerances and allergies. So, the fact that microbiome markers almost always outperformed genomic SNP markers may be due to the markedly different dataset sizes. Third, inclusion criteria in this study did not consider factors known to influence the microbiome composition (probiotic or antibiotic usage) or other comorbidities (musculoskeletal pain, skin conditions, hypothyroidism, diabetes, cholesterol, hypertension, and mental health) that may confound the results presented. Fourth, the survey instrument utilized was an *ad hoc* questionnaire that asked participants to rate their symptom severity for different FGIDs on a scale of 1–5 and was not a validated clinical instrument. The survey was performed retrospectively for both time points after subjects successfully achieved 5% or more body weight loss. And fifth, the findings from this study are derived from a weight loss cohort and thus may be only reflective of the population with FGID that is overweight or obese, or that may benefit from weight loss.

## Conclusion

Despite the above limitations, the digital therapeutics care provided to subjects, informed by genetic and baseline gut microbiome and their interaction with participant’s lifestyle, effectively reduced symptom severity of FGIDs, including IBS, diarrhea, and constipation. One of our earlier studies supported the use of this care as a therapy for insulin resistance (Ricchetti et al., 2020), empowering subjects to manage their inflammation by awareness of the impact of processed foods and foods to which they are sensitive as per their genomic SNPs and gut microbiome results. Dietary fiber coaching also resulted in increased vegetable diversity and quantity. Whereas further research is required to better understand the effect of different components of the care (e.g., fiber types) on modulating the microbial taxa and genomic SNPs identified in the models and their corresponding effect on reduction of FGIDs symptom severity, this preliminary retrospective study generates testable hypotheses for associations of several biomarkers with FGID status and with the prognosis of FGID symptomatology. This study thus provides proof of concept on how a combined genetic and gut microbiome-based dietary intervention can yield biomarkers from human studies. Moreover, the methods presented add to the existing set of tools (e.g., Polster et al., 2021) that can be readily implemented to understand the role that genetics and gut microbiome play on disease etiology. Additionally, FGID and overweight or obesity are common comorbidities, yet concomitant reduction of body weight and reduction of FGID symptom severity is an endpoint which has been poorly studied but it is of high interest to clinicians and patients.

## Data Availability Statement

The datasets presented in this study can be found in online repositories. The names of the repository/repositories and accession number(s) can be found below: https://www.ncbi.nlm.nih.gov/bioproject/PRJNA760529.

## Ethics Statement

The studies involving human participants were reviewed and approved by Ethical and Independent Review Services (E&I). The patients/participants provided their written informed consent to participate in this study.

## Author Contributions

SK: formal analysis, methodology, software, visualization, writing – original draft, and writing – review and editing. PF-L: conceptualization, data curation, formal analysis, methodology, software, validation, visualization, writing – original draft, and writing – review and editing. DK: data curation, formal analysis, methodology, software, visualization, and writing – original draft. TU and KM: data curation and software. CI and RR: writing – original draft and writing – review and editing. SS-R and DA: project administration, writing – original draft, and writing – review and editing. JU: software and writing- reviewing. PD: writing – review and editing. RS: conceptualization, funding acquisition, and writing – review and editing. All authors contributed to the article and approved the submitted version.

## Conflict of Interest

All authors except JU and PD were employees of Digbi Health. JU did contractual work for Digbi Health. PD was an advisor to Digbi Health. The authors declare that this study received funding from Digbi Health. The funder Digbi Health was not involved in the study design, collection, analysis, interpretation of data, the writing of this article, or the decision to submit it for publication. SK, DA, and RS had a patent-pending concerning this work: US Application No. 63/246,348, Methods and systems for multi-omic interventions as diagnostics for personalized care of functional gastrointestinal disorders. The digital therapeutics program provided to study participants in this work is a commercially available program developed and marketed by Digbi Health.

## Publisher’s Note

All claims expressed in this article are solely those of the authors and do not necessarily represent those of their affiliated organizations, or those of the publisher, the editors and the reviewers. Any product that may be evaluated in this article, or claim that may be made by its manufacturer, is not guaranteed or endorsed by the publisher.

## References

[B1] Agakidis C. Kotzakioulafi E. Petridis D. Apostolidou K. Karagiozoglou-Lampoudi T. (2019). Mediterranean diet adherence is associated with lower prevalence of functional gastrointestinal disorders in children and adolescents. *Nutrients* 11:1283. 10.3390/nu11061283 PMC6628326 31174310

[B2] Agnello M. Carroll L. N. Imam N. Pino R. Palmer C. Varas I. (2020). Gut microbiome composition and risk factors in a large cross-sectional IBS cohort. *BMJ Open Gastroenterol.* 7:e000345. 10.1136/bmjgast-2019-000345 32518661 PMC7254124

[B3] American College of Gastroenterology (2021). *10 Tips on Belching, Bloating, and Flatulence.* Available online at: https://gi.org/topics/digestive-health-tips/ (accessed September 21, 2021).

[B4] Basnayake C. Kamm M. A. Salzberg M. Stanley A. Khera A. Burrell K. (2019). Outcome of hospital outpatient treatment of functional gastrointestinal disorders: functional gastrointestinal disorders. *Intern. Med. J.* 49 225–231. 10.1111/imj.14067 30091176

[B5] Beyder A. Mazzone A. Strege P. R. Tester D. J. Saito Y. A. Bernard C. E. (2014). Loss-of-function of the voltage-gated sodium channel NaV1.5 (Channelopathies) in patients with irritable bowel syndrome. *Gastroenterology* 146 1659–1668. 10.1053/j.gastro.2014.02.054 24613995 PMC4096335

[B6] Black C. J. Drossman D. A. Talley N. J. Ruddy J. Ford A. C. (2020). Functional gastrointestinal disorders: advances in understanding and management. *Lancet* 396 1664–1674. 10.1016/S0140-6736(20)32115-2 33049221

[B7] Bolyen E. Rideout J. R. Dillon M. R. Bokulich N. A. Abnet C. C. Al-Ghalith G. A. (2019). Reproducible, interactive, scalable and extensible microbiome data science using QIIME 2. *Nat. Biotechnol.* 37 852–857. 10.1038/s41587-019-0209-9 31341288 PMC7015180

[B8] Bui T. P. N. Troise A. D. Nijsse B. Roviello G. N. Fogliano V. de Vos W. M. (2020). Intestinimonas-like bacteria are important butyrate producers that utilize Nε-fructosyllysine and lysine in formula-fed infants and adults. *J. Funct. Foods* 70:103974. 10.1016/j.jff.2020.103974

[B9] Buono J. L. Mathur K. Averitt A. J. Andrae D. A. (2017). Economic burden of irritable bowel syndrome with diarrhea: retrospective analysis of a U.S. Commercially Insured Population. *J. Manag. Care Spec. Pharm.* 23 453–460. 10.18553/jmcp.2016.16138 28345443 PMC10398241

[B10] Carco C. Young W. Gearry R. B. Talley N. J. McNabb W. C. Roy N. C. (2020). Increasing evidence that irritable bowel syndrome and functional gastrointestinal disorders have a microbial pathogenesis. *Front. Cell. Infect. Microbiol.* 10:468. 10.3389/fcimb.2020.00468 33014892 PMC7509092

[B11] Chang L. (2004). Review article: epidemiology and quality of life in functional gastrointestinal disorders. *Aliment. Pharmacol. Ther.* 20 31–39. 10.1111/j.1365-2036.2004.02183.x 15521853

[B12] Chen H. Mao X. He J. Yu B. Huang Z. Yu J. (2013). Dietary fibre affects intestinal mucosal barrier function and regulates intestinal bacteria in weaning piglets. *Br. J. Nutr.* 110 1837–1848. 10.1017/S0007114513001293 23656640

[B13] Clouse R. E. Mayer E. A. Aziz Q. Drossman D. A. Dumitrascu D. L. Mönnikes H. (2006). Functional abdominal pain syndrome. *Gastroenterology* 130 1492–1497. 10.1053/j.gastro.2005.11.062 16678562

[B14] Cruz-Aguliar R. M. Wantia N. Clavel T. Vehreschild M. J. G. T. Buch T. Bajbouj M. (2019). An open-labeled study on fecal microbiota transfer in irritable bowel syndrome patients reveals improvement in abdominal pain associated with the relative abundance of akkermansia muciniphila. *Digestion* 100 127–138. 10.1159/000494252 30423561

[B15] Dellon E. S. Ringel Y. (2006). Treatment of functional diarrhea. *Curr. Treat. Options Gastroenterol.* 9 331–342. 16836952 10.1007/s11938-006-0015-6

[B16] Derrien M. van Passel M. W. J. van de Bovenkamp J. H. B. Schipper R. de Vos W. Dekker J. (2010). Mucin-bacterial interactions in the human oral cavity and digestive tract. *Gut Microbes* 1 254–268. 10.4161/gmic.1.4.12778 21327032 PMC3023607

[B17] Elli L. Tomba C. Branchi F. Roncoroni L. Lombardo V. Bardella M. (2016). Evidence for the presence of non-celiac gluten sensitivity in patients with functional gastrointestinal symptoms: results from a multicenter randomized double-blind placebo-controlled gluten challenge. *Nutrients* 8:84. 10.3390/nu8020084 26867199 PMC4772047

[B18] Emerenziani S. Pier Luca Guarino M. Trillo Asensio L. Altomare A. Ribolsi M. Balestrieri P. (2019). Role of overweight and obesity in gastrointestinal disease. *Nutrients* 12:111. 10.3390/nu12010111 PMC7019431 31906216

[B19] Enroth S. Johansson Å Enroth S. B. Gyllensten U. (2014). Strong effects of genetic and lifestyle factors on biomarker variation and use of personalized cutoffs. *Nat. Commun.* 5:4684. 10.1038/ncomms5684 25147954 PMC4143927

[B20] Everhart J. E. (2008). “All digestive diseases,” in *The Burden of Digestive Diseases in the United States*, ed. Everhart J. E. (Washington, DC: US Government Printing Office), 1–6.

[B21] Fernandes A. D. Reid J. N. Macklaim J. M. McMurrough T. A. Edgell D. R. Gloor G. B. (2014). Unifying the analysis of high-throughput sequencing datasets: characterizing RNA-seq, 16S rRNA gene sequencing and selective growth experiments by compositional data analysis. *Microbiome* 2:15. 10.1186/2049-2618-2-15 24910773 PMC4030730

[B22] Friedman J. Hastie T. Tibshirani R. (2010). Regularization paths for generalized linear models via coordinate descent. *J. Stat. Softw.* 33 1–22. 10.18637/jss.v033.i01 20808728 PMC2929880

[B23] Fryar C. D. Carroll M. D. Afful J. (2020). *Prevalence of Overweight, Obesity, and Severe Obesity Among Adults Aged 20 and Over: United States, 1960–1962 Through 2017–2018. NCHS Health E-Stats. 2020.* Available online at: https://www.cdc.gov/nchs/data/hestat/obesity-adult-17-18/obesity-adult.htm (accessed September 10, 2021).

[B24] Galai T. Moran-Lev H. Cohen S. Ben-Tov A. Levy D. Weintraub Y. (2020). Higher prevalence of obesity among children with functional abdominal pain disorders. *BMC Pediatr.* 20:193. 10.1186/s12887-020-2106-2109 PMC7201594 32375714

[B25] Gloor G. B. Macklaim J. M. Pawlowsky-Glahn V. Egozcue J. J. (2017). Microbiome datasets are compositional: and this is not optional. *Front. Microbiol.* 8:2224. 10.3389/fmicb.2017.02224 29187837 PMC5695134

[B26] Greenacre M. (2018). *Compositional Data Analysis in Practice.* New York, NY: Chapman and Hall/CRC.

[B27] Greenacre M. Martínez-Álvaro M. Blasco A. (2021). Compositional data analysis of microbiome and any-omics datasets: a validation of the additive logratio transformation. *Front. Microbiol.* 12:727398. 10.3389/fmicb.2021.727398 34737726 PMC8561721

[B28] Hasler W. L. (2006). Gas and bloating. *Gastroenterol. Hepatol.* 2 654–662. PMC5350578 28316536

[B29] Henström M. D’Amato M. (2016). Genetics of irritable bowel syndrome. *Mol. Cell. Pediatr.* 3:7. 10.1186/s40348-016-0038-6 26873717 PMC4752571

[B30] Ho W. Spiegel B. M. (2008). The relationship between obesity and functional gastrointestinal disorders: causation, association, or neither? *Gastroenterol. Hepatol.* 4 572–578. PMC3096111 21960939

[B31] Illumina (2013). *Illumina documentation, Part # 15044223 Rev. B*. Available online at: https://support.illumina.com/content/dam/illumina-support/documents/documentation/chemistry_documentation/16s/16s-metagenomic-library-prep-guide-15044223-b.pdf (accessed September 21, 2021).

[B32] International Foundation for Gastrointestinal Disorders (2021). *About GI Motility.* Available online at: https://aboutgimotility.org/learn-about-gi-motility/ (accessed September, 2021).

[B33] Jin M. Kalainy S. Baskota N. Chiang D. Deehan E. C. McDougall C. (2019). Faecal microbiota from patients with cirrhosis has a low capacity to ferment non-digestible carbohydrates into short-chain fatty acids. *Liver Int.* 39 1437–1447. 10.1111/liv.14106 30919578

[B34] Johannesson E. Ringström G. Abrahamsson H. Sadik R. (2015). Intervention to increase physical activity in irritable bowel syndrome shows long-term positive effects. *World J. Gastroenterol.* 21 600–608. 10.3748/wjg.v21.i2.600 25593485 PMC4294172

[B35] Johannesson E. Simrén M. Strid H. Bajor A. Sadik R. (2011). Physical activity improves symptoms in irritable bowel syndrome: a randomized controlled trial. *Am. J. Gastroenterol.* 106 915–922. 10.1038/ajg.2010.480 21206488

[B36] Bisanz Jordan E (2018). *qiime2R**: Importing QIIME2 Artifacts and Associated Data into R Sessions.* Available online at: https://github.com/jbisanz/qiime2R (accessed July 10, 2021).

[B37] Kennedy P. J. (2014). Irritable bowel syndrome: a microbiome-gut-brain axis disorder? *World J. Gastroenterol.* 20:14105. 10.3748/wjg.v20.i39.14105 25339800 PMC4202342

[B38] Kim H. I. Jung S. A. Choi J. Y. Kim S. E. Jung H. K. Shim K. N. (2013). Impact of shiftwork on irritable bowel syndrome and functional dyspepsia. *J. Korean Med. Sci.* 28 431–437. 10.3346/jkms.2013.28.3.431 23487413 PMC3594608

[B39] Kim Y. S. Unno T. Kim B.-Y. Park M.-S. (2020). Sex differences in gut microbiota. *World J. Mens. Health* 38:48. 10.5534/wjmh.190009 30929328 PMC6920072

[B40] Kushkevych I. Dordević D. Kollar P. Vítězová M. Drago L. (2019). Hydrogen sulfide as a toxic product in the small–large intestine axis and its role in IBD development. *J. Clin. Med.* 8:1054. 10.3390/jcm8071054 PMC6679076 31330956

[B41] Lee S. H. You H. S. Kang H.-G. Kang S. S. Hyun S. H. (2020). Association between altered blood parameters and gut microbiota after synbiotic intake in healthy, elderly korean women. *Nutrients* 12:3112. 10.3390/nu12103112 33053824 PMC7650560

[B42] Lembo A. Zaman M. Jones M. Talley N. J. (2007). Influence of genetics on irritable bowel syndrome, gastro-oesophageal reflux and dyspepsia: a twin study: influence of genetics on ibs, gerd and dyspepsia. *Aliment. Pharmacol. Ther.* 25 1343–1350. 10.1111/j.1365-2036.2007.03326.x 17509102

[B43] Locke G. R. Ackerman M. J. Zinsmeister A. R. Thapa P. Farrugia G. (2006). Gastrointestinal symptoms in families of patients with an SCN5A-encoded cardiac channelopathy: evidence of an intestinal channelopathy. *Am. J. Gastroenterol.* 101 1299–1304. 10.1111/j.1572-0241.2006.00507.x 16771953

[B44] Longstreth G. F. Thompson W. G. Chey W. D. Houghton L. A. Mearin F. Spiller R. C. (2006). Functional bowel disorders. *Gastroenterology* 130 1480–1491. 10.1053/j.gastro.2005.11.061 16678561

[B45] Luu M. Riester Z. Baldrich A. Reichardt N. Yuille S. Busetti A. (2021). Microbial short-chain fatty acids modulate CD8+ T cell responses and improve adoptive immunotherapy for cancer. *Nat. Commun.* 12:4077. 10.1038/s41467-021-24331-1 34210970 PMC8249424

[B46] Lyra A. Rinttilä T. Nikkilä J. Krogius-Kurikka L. Kajander K. Malinen E. (2009). Diarrhoea-predominant irritable bowel syndrome distinguishable by 16S rRNA gene phylotype quantifcation. *World J. Gastroenterol.* 15:5936. 10.3748/wjg.15.5936 20014457 PMC2795180

[B47] Manning L. P. Biesiekierski J. R. (2018). Use of dietary interventions for functional gastrointestinal disorders. *Curr. Opin. Pharmacol.* 43 132–138. 10.1016/j.coph.2018.09.003 30308416

[B48] Marsh A. Eslick E. M. Eslick G. D. (2016). Does a diet low in FODMAPs reduce symptoms associated with functional gastrointestinal disorders? A comprehensive systematic review and meta-analysis. *Eur. J. Nutr.* 55 897–906. 10.1007/s00394-015-0922-1 25982757

[B49] McFarland L. V. Goh S. (2019). Are probiotics and prebiotics effective in the prevention of travellers’ diarrhea: a systematic review and meta-analysis. *Travel Med. Infect. Dis.* 27 11–19. 10.1016/j.tmaid.2018.09.007 30278238

[B50] McMurdie P. J. Holmes S. (2013). phyloseq: an R package for reproducible interactive analysis and graphics of microbiome census data. *PLoS One* 8:e61217. 10.1371/journal.pone.0061217 23630581 PMC3632530

[B51] Monsuur A. J. de Bakker P. I. W. Zhernakova A. Pinto D. Verduijn W. Romanos J. (2008). Effective detection of human leukocyte antigen risk alleles in celiac disease using tag single nucleotide polymorphisms. *PLoS One* 3:e2270. 10.1371/journal.pone.0002270 18509540 PMC2386975

[B52] Morito Y. Aimi M. Ishimura N. Shimura S. Mikami H. Okimoto E. (2014). Association between sleep disturbances and abdominal symptoms. *Intern. Med.* 53 2179–2183. 10.2169/internalmedicine.53.2591 25274228

[B53] Narayanan S. P. Anderson B. Bharucha A. E. (2021). Sex- and gender-related differences in common functional gastroenterologic disorders. *Mayo Clin. Proc.* 96 1071–1089. 10.1016/j.mayocp.2020.10.004 33814075 PMC8075061

[B54] Ohkusa T. Koido S. Nishikawa Y. Sato N. (2019). Gut microbiota and chronic constipation: a review and update. *Front. Med.* 6:19. 10.3389/fmed.2019.00019 30809523 PMC6379309

[B55] Palarea-Albaladejo J. Martín-Fernández J. A. (2015). zCompositions — R package for multivariate imputation of left-censored data under a compositional approach. *Chemom. Intell. Lab. Syst.* 143 85–96. 10.1016/j.chemolab.2015.02.019

[B56] Peery A. F. Crockett S. D. Murphy C. C. Lund J. L. Dellon E. S. Williams J. L. (2019). Burden and cost of gastrointestinal, liver, and pancreatic diseases in the United States: update 2018. *Gastroenterology* 156 254.e11–272.e11. 10.1053/j.gastro.2018.08.063 30315778 PMC6689327

[B57] Polster A. Öhman L. Tap J. Derrien M. Le Nevé B. Sundin J. (2021). A novel stepwise integrative analysis pipeline reveals distinct microbiota-host interactions and link to symptoms in irritable bowel syndrome. *Sci. Rep.* 11:5521. 10.1038/s41598-021-84686-9 33750831 PMC7943560

[B58] Quinn T. P. Erb I. Gloor G. Notredame C. Richardson M. F. Crowley T. M. (2019). A field guide for the compositional analysis of any-omics data. *GigaScience* 8:giz107. 10.1093/gigascience/giz107 31544212 PMC6755255

[B59] Ricchetti R. R. Sinha R. Muthukumar K. Singh-Rambiritch S. Underwood B. Junaid I. (2020). Outcomes of a precision digital care program for obesity and associated comorbidities: results in real world clinical practice. *Int. J. Clin. Med. Cases* 7:160.

[B60] Saffouri G. B. Shields-Cutler R. R. Chen J. Yang Y. Lekatz H. R. Hale V. L. (2019). Small intestinal microbial dysbiosis underlies symptoms associated with functional gastrointestinal disorders. *Nat. Commun.* 10:2012. 10.1038/s41467-019-9964-9967 PMC6494866 31043597

[B61] Saito Y. A. Petersen G. M. Larson J. J. Atkinson E. J. Fridley B. L. de Andrade M. (2010). Familial aggregation of irritable bowel syndrome: a family case–control study. *Am. J. Gastroenterol.* 105 833–841. 10.1038/ajg.2010.116 20234344 PMC2875200

[B62] Saito Y. A. Strege P. R. Tester D. J. Locke G. R. Talley N. J. Bernard C. E. (2009). Sodium channel mutation in irritable bowel syndrome: evidence for an ion channelopathy. *Am. J. Physiol. Gastrointest. Liver Physiol.* 296 G211–G218. 10.1152/ajpgi.90571.2008 19056759 PMC2643921

[B63] Schnabel L. Buscail C. Sabate J.-M. Bouchoucha M. Kesse-Guyot E. Allès B. (2018). Association between ultra-processed food consumption and functional gastrointestinal disorders: results from the french nutrinet-santé cohort. *Am. J. Gastroenterol.* 113 1217–1228. 10.1038/s41395-018-0137-1 29904158

[B64] Shau J.-P. Chen P.-H. Chan C.-F. Hsu Y.-C. Wu T.-C. James F. E. (2016). Fast foods - are they a risk factor for functional gastrointestinal disorders? *Asia Pac. J. Clin. Nutr.* 25 393–401. 27222424 10.6133/apjcn.2016.25.2.28

[B65] Silva 138 Reference database (2021). Available online at: https://www.arb-silva.de/documentation/release-138/ (accessed September 10, 2021).

[B66] Simrén M. Barbara G. Flint H. J. Spiegel B. M. R. Spiller R. C. Vanner S. (2013). Intestinal microbiota in functional bowel disorders: a Rome foundation report. *Gut* 62 159–176. 10.1136/gutjnl-2012-302167 22730468 PMC3551212

[B67] Singh S. Lin H. (2015). Hydrogen sulfide in physiology and diseases of the digestive tract. *Microorganisms* 3 866–889. 10.3390/microorganisms3040866 27682122 PMC5023273

[B68] Sinha R. Kachru D. Ricchetti R. R. Singh-Rambiritch S. Muthukumar K. M. Singaravel V. (2021). Leveraging genomic associations in precision digital care for weight loss: cohort study. *J. Med. Internet Res.* 23:e25401. 10.2196/25401 33849843 PMC8173391

[B69] SNPedia database (2021). Available online at: https://www.snpedia.com/index.php/Rs1800896 (accessed September 10, 2021).

[B70] Sperber A. D. Bangdiwala S. I. Drossman D. A. Ghoshal U. C. Simren M. Tack J. (2021). Worldwide prevalence and burden of functional gastrointestinal disorders, results of rome foundation global study. *Gastroenterology* 160 99.e13–114.e13. 10.1053/j.gastro.2020.04.014 32294476

[B71] Sullivan S. N. (2012). Functional abdominal bloating with distention. *ISRN Gastroenterol.* 2012:721820. 10.5402/2012/721820 22778978 PMC3388350

[B72] Takeshita K. Mizuno S. Mikami Y. Sujino T. Saigusa K. Matsuoka K. (2016). A single species of clostridium subcluster XIVa decreased in ulcerative colitis patients. *Inflamm. Bowel Dis.* 22 2802–2810. 10.1097/MIB.0000000000000972 27824645

[B73] Tan J. McKenzie C. Potamitis M. Thorburn A. N. Mackay C. R. Macia L. (2014). “The role of short-chain fatty acids in health and disease,” in *Advances in Immunology*, ed. Alt F. W. (Amsterdam: Elsevier), 91–119. 10.1016/B978-0-12-800100-4.00003-9 24388214

[B74] Thompson W. G. Longstreth G. F. Drossman D. A. Heaton K. W. Irvine E. J. Muller-Lissner S. A. (1999). Functional bowel disorders and functional abdominal pain. *Gut* 45 ii43–ii47. 10.1136/gut.45.2008.ii43 10457044 PMC1766683

[B75] Van Heel D. A. Franke L. Hunt K. A. Gwilliam R. Zhernakova A. Inouye M. (2007). A genome-wide association study for celiac disease identifies risk variants in the region harboring IL2 and IL21. *Nat. Genet.* 39 827–829. 10.1038/ng2058 17558408 PMC2274985

[B76] Wei L. Singh R. Ro S. Ghoshal U. C. (2021). Gut microbiota dysbiosis in functional gastrointestinal disorders: underpinning the symptoms and pathophysiology. *JGH Open* 5 976–987. 10.1002/jgh3.12528 34584964 PMC8454481

[B77] Wilder-Smith C. H. Olesen S. S. Materna A. Drewes A. M. (2017). Predictors of response to a low-FODMAP diet in patients with functional gastrointestinal disorders and lactose or fructose intolerance. *Aliment. Pharmacol. Ther.* 45 1094–1106. 10.1111/apt.13978 28233394

[B78] Zhao Y. Yu Y.-B. (2016). Intestinal microbiota and chronic constipation. *SpringerPlus* 5:1130. 10.1186/s40064-016-2821-1 27478747 PMC4951383

