# Supplementary material for: Digital Therapeutics Care Utilizing Genetic and Gut Microbiome Signals for the Management of Functional Gastrointestinal Disorders: Results From a Preliminary Retrospective Study
Source: Front Microbiol. 2022 Mar 21;13:826916. doi: 10.3389/fmicb.2022.826916 (PMC8983270; doi:10.3389/fmicb.2022.826916)
Supplement: Supplementary file 1 [file Data_Sheet_1.PDF]

Supplementary Material

**Supplementary Table 1.** Genome SNPs utilized in this work.

| Trait | rsID | Gene | Risk Allele | References |
|---|---|---|---|---|
| Caffeine Metabolism | rs2472297 | CYP1A2 | C | https://pubmed.ncbi.nlm.nih.gov/27702941/ |
| | rs762551 | CYP1A2 | C | https://pubmed.ncbi.nlm.nih.gov/18398030/ |
| Gluten Sensitivity | rs2187668 | HLA-DQ 2.5 | T | https://pubmed.ncbi.nlm.nih.gov/20190752/ |
| | rs2395182 | HLA-DQ 2.2 (M1) | T | https://pubmed.ncbi.nlm.nih.gov/27702941/ |
| | rs4639334 | HLA-DQ7 | A | https://pubmed.ncbi.nlm.nih.gov/18509540/ |
| | rs4713586 | HLA-DQ 2.2 (M3) | G | https://www.ncbi.nlm.nih.gov/pmc/articles/PMC2386975/ |
| | rs7454108 | HLA-DQ8 | C | https://www.ncbi.nlm.nih.gov/pmc/articles/PMC2386975/ |
| | rs7775228 | HLA-DQ 2.2 | C | https://www.ncbi.nlm.nih.gov/pmc/articles/PMC2386975/ |
| Lactose Intolerance | rs182549 | MCM6 | C | https://pubmed.ncbi.nlm.nih.gov/28588231/ |
| | rs4988235 | MCM6 | G | https://pubmed.ncbi.nlm.nih.gov/15114531/ |
| Milk Allergy | rs324015 | STAT6 | T | https://pubmed.ncbi.nlm.nih.gov/23535094/ |
| Peanut Allergy | rs7192 | HLA-DRA | T | https://pubmed.ncbi.nlm.nih.gov/25710614/ |
| | rs9275596 | MTCO3P1 - AL662789.1 | C | https://pubmed.ncbi.nlm.nih.gov/25710614/ |
| Inflammatory Markers | rs1800629 | TNF | A | https://pubmed.ncbi.nlm.nih.gov/15803022/ |
| | rs1800896 | IL10 | T | https://pubmed.ncbi.nlm.nih.gov/29310417/ |
| | rs3024496 | IL10 | G | https://pubmed.ncbi.nlm.nih.gov/24628819/ |

**Supplementary Table 2.** PERMANOVA analysis for baseline gut microbiome of subjects considering FGID status, BMI, gender, and interactions FGID status:BMI and FGID status:gender as covariates.

| | Df | Sums Of Sqs | Mean Sqs | F. Model | $R^2$ | *P* value |
|---|---|---|---|---|---|---|
| FGID status | 1 | 0.00826 | 0.0082617 | 0.88650 | 0.00534 | 0.532 |
| BMI | 1 | 0.01056 | 0.0105565 | 1.13274 | 0.00682 | 0.279 |
| Gender | 1 | 0.01855 | 0.0185488 | 1.99034 | 0.01198 | *0.030*[a] |
| FGID status:BMI | 1 | 0.00610 | 0.0061003 | 0.65458 | 0.00394 | 0.819 |
| FGID status:Gender | 1 | 0.01387 | 0.0138673 | 1.48801 | 0.00896 | 0.102 |
| Residuals | 160 | 1.49111 | 0.0093194 | | 0.96297 | |
| Total | 165 | 1.54844 | | | 1.00000 | |

[a]Values in italics are significant (*P*=<0.05)

**Supplementary Table 3.** Reduction in Summative FGID Symptom Severity Linear Model: Demographics + Genomics (D+G)

| Variable | Estimate | Std. Error | t value | *P* value |
|---|---|---|---|---|
| Gluten Sensitivity (rs4639334), Risk Allele A | -2.839 | 0.896 | -3.167 | *0.002*[a] |
| Gluten Sensitivity (rs7775228), Risk Allele C | -2.763 | 0.975 | -2.835 | *0.006* |

Adjusted $R^2$: 0.124
[a]Values in italics are significant (*P*=<0.05)

**Supplementary Table 4.** Reduction in Summative FGID Symptom Severity Linear Model: Demographics + Microbiome (D+M)

| Variable | Estimate | Std. Error | t value | *P* value |
|---|---|---|---|---|
| *Ruminococcus torques* group | 0.727 | 0.250 | 2.915 | *0.004*[a] |
| Candidatus *Soleaferrea* | -0.359 | 0.146 | -2.463 | *0.016* |
| *Intestinimonas* | 0.340 | 0.120 | 2.843 | *0.006* |
| Unclassified genus GCA-900066575 of *Lachnospiraceae* family | 0.474 | 0.143 | 3.307 | *0.001* |
| *Eubacterium hallii group* | -0.366 | 0.137 | -2.667 | *0.009* |
| *Alistipes* | -0.358 | 0.139 | -2.578 | *0.012* |
| *Megasphaera* | 0.415 | 0.132 | 3.154 | *0.002* |
| *Desulfovibrio* | -0.395 | 0.114 | -3.461 | *0.001* |

Adjusted $R^2$: 0.318
[a]Values in italics are significant (*P*=<0.05)

**Supplementary Table 5.** Reduction in IBS Symptom Severity Linear Model: Demographics + Genomics (D+G)

| Variable | Estimate | Std. Error | t value | *P* value |
|---|---|---|---|---|
| Gluten Sensitivity (rs7775228), Risk Allele C | -0.829 | 0.276 | -3.009 | *0.004*[a] |

Adjusted $R^2$: 0.130
[a]Values in italics are significant ($P$=<0.05)

**Supplementary Table 6.** Reduction in IBS Symptom Severity Linear Model: Demographics + Microbiome (D+M)

| Variable | Estimate | Std. Error | t value | *P* value |
|---|---|---|---|---|
| *Unclassified genus Clostridia UCG-014* | 0.115 | 0.026 | 4.491 | *<0.001*[a] |
| *Escherichia-Shigella* | 0.111 | 0.028 | 3.982 | *<0.001* |
| *Fusicatenibacter* | -0.108 | 0.039 | -2.753 | *0.008* |
| *Tyzzerella* | -0.098 | 0.031 | -3.143 | *0.003* |
| *Megasphaera* | 0.107 | 0.033 | 3.244 | *0.002* |
| *Moryella* | -0.089 | 0.029 | -3.088 | *0.003* |

Adjusted $R^2$: 0.432
[a]Values in italics are significant ($P$=<0.05)

**Supplementary Table 7.** Reduction in Constipation Symptom Severity Linear Model: Demographics + Genomics (D+G)

| Variable | Estimate | Std. Error | t value | *P* value |
|---|---|---|---|---|
| Gluten Sensitivity (rs4639334), Risk Allele A | -0.558 | 0.286 | -1.948 | 0.056 |

Adjusted $R^2$: 0.038

**Supplementary Table 8.** Reduction in Constipation Symptom Severity Linear Model: Demographics + Microbiome (D+M)

| Variable | Estimate | Std. Error | t value | *P* value |
|---|---|---|---|---|
| *Parabacteroides* | 0.157 | 0.073 | 2.167 | *0.036*[a] |
| *Eubacterium coprostanoligenes* group | -0.227 | 0.057 | -3.944 | *<0.001* |
| Unclassified genus of *Anaerovoracaceae* Family XIII AD3011 group | 0.183 | 0.070 | 2.609 | *0.013* |
| *Lachnospira* | 0.101 | 0.047 | 2.162 | *0.037* |
| *Terrisporobacter* | 0.081 | 0.040 | 2.024 | 0.050 |

Adjusted $R^2$: 0.413
[a]Values in italics are significant ($P$=<0.05)

**Supplementary Table 9.** Reduction in Diarrhea Symptom Severity Linear Model: Demographics + Genomics (D+G)

| **Variable** | **Estimate** | **Std. Error** | **t value** | ***P* value** |
|---|---|---|---|---|
| Gluten Sensitivity (rs7775228), Risk Allele C | -0.773 | 0.330 | -2.339 | *0.024*[a] |

Adjusted $R^2$: 0.090
[a]Values in italics are significant (*P*=<0.05)

**Supplementary Table 10.** Reduction in Diarrhea Symptom Severity Linear Model: Demographics + Microbiome (D+M)

| **Variable** | **Estimate** | **Std. Error** | **t value** | ***P* value** |
|---|---|---|---|---|
| *Intestinimonas* | 0.092 | 0.032 | 2.857 | *0.008*[a] |
| *Prevotella* | 0.091 | 0.027 | 3.365 | *0.002* |
| Unclassified genus UCG-009 of *Butyricicoccaceae* family | -0.129 | 0.031 | -4.182 | *<0.001* |
| *Lactobacillus* | 0.096 | 0.037 | 2.608 | *0.015* |
| *Phascolarctobacterium* | 0.059 | 0.026 | 2.260 | *0.032* |

Adjusted $R^2$: 0.610
[a]Values in italics are significant (*P*=<0.05)
